# Prognostic value of CTI for major adverse cardiovascular events in patients With ST-elevation myocardial infarction after primary percutaneous coronary intervention

**DOI:** 10.3389/fcvm.2026.1832830

**Published:** 2026-05-26

**Authors:** Qiang Zhao, Shenwen Fu, Xiaokang Hu, Xianqing Hu

**Affiliations:** 1 Department of Cardiovascular Medicine, Jinhua Municipal Central Hospital, Jinhua Hospital of Zhejiang University, Jinhua, Zhejiang, China; 2 Vascular Signalling, Molecular Cardiology, Department of Cardiology I-Coronary and Peripheral Vascular Disease, Heart Failure, University Hospital Münster, Münster, Germany

**Keywords:** acute heart failure, C-reactive protein–triglyceride glucose index, major adverse cardiovascular events, percutaneous coronary intervention, ST-segment elevation myocardial infarction

## Abstract

**Methods:**

A total of 618 STEMI patients who underwent PCI were retrospectively enrolled. The primary endpoint was 1-year MACE. Feature selection was performed using the Boruta algorithm. Multivariable Cox regression analysis was conducted to evaluate the independent association between CTI and MACE. The incremental prognostic information provided by CTI was assessed using 365-day time-dependent receiver operating characteristic (ROC) curves and the DeLong test, while clinical utility was evaluated using decision curve analysis (DCA). Subgroup analysis was performed according to diabetes status. Sensitivity analyses using different adjustment strategies were conducted to assess the robustness of the results.

**Results:**

During follow-up, 139 patients (22.5%) experienced MACE. CTI was identified as an important predictor by the Boruta algorithm and remained independently associated with MACE in multivariable Cox regression analysis (HR = 1.443, 95% CI: 1.191–1.748, *P* < 0.001). Although the addition of CTI to the baseline model resulted in only modest improvement in discrimination and clinical net benefit, further component analysis showed that the association between CTI and MACE was mainly driven by acute heart failure events. Subgroup analysis demonstrated consistent associations between CTI and MACE regardless of diabetes status (*P* for interaction = 0.504). The association between CTI and MACE remained robust across multiple sensitivity analyses.

**Conclusions:**

CTI was an independent predictor of MACE in STEMI patients undergoing PCI. Notably, the association appeared to be mainly driven by acute heart failure events, suggesting a close relationship between CTI and post-infarction ventricular dysfunction.

## 1 Introduction

ST-segment elevation myocardial infarction (STEMI) is one of the most severe manifestations of coronary artery disease and remains a major cause of morbidity and mortality worldwide. Percutaneous coronary intervention (PCI) has become the preferred reperfusion strategy for patients with STEMI and significantly improves survival (1, 2). However, despite advances in interventional techniques and pharmacological therapy, a considerable proportion of patients continue to experience major adverse cardiovascular events (MACE) after PCI, particularly those who are elderly or have comorbidities such as diabetes—the incidence of adverse cardiovascular events remains relatively high, reaching approximately 20% (3). Currently, risk prediction for patients with STEMI mainly relies on traditional models based on clinical characteristics, imaging findings, and comorbid conditions (4, 5). Nevertheless, the predictive performance of several classical biochemical biomarkers remains limited, which may restrict accurate risk stratification in clinical practice (6–8). Therefore, identifying novel and reliable biomarkers to improve risk assessment and prognostic evaluation in patients with STEMI is of considerable clinical importance (9).

STEMI is considered a metabolically influenced disease characterized by significant alterations in metabolic products. Inflammatory responses, oxidative stress, and immune activation jointly contribute to the initiation and progression of STEMI by promoting endothelial dysfunction, plaque instability, and thrombus formation. Metabolic disturbances such as hyperglycemia and dyslipidemia can further aggravate vascular inflammation and oxidative injury, thereby accelerating atherosclerotic processes. A large body of evidence has demonstrated that patients with elevated blood glucose levels and heightened systemic inflammation have a significantly higher incidence of adverse cardiovascular events (10–12). Therefore, biomarkers that integrate metabolic and inflammatory status may provide additional value in risk stratification and prognostic evaluation for patients with STEMI.

The C-reactive protein–triglyceride glucose index (CTI) was first proposed by Ruan et al. as a composite biomarker that comprehensively reflects both insulin resistance and systemic inflammatory status (13). Among its components, the triglyceride–glucose (TyG) index, calculated from fasting triglycerides (TG) and fasting blood glucose (FBG), has been widely recognized as a simple and reliable surrogate marker of insulin resistance and has demonstrated significant value in cardiovascular risk stratification and outcome prediction (14). On this basis, CTI further incorporates C-reactive protein (CRP), a well-established and powerful marker of systemic inflammation, thereby integrating metabolic and inflammatory information into a single indicator (15). Recent evidence suggests that elevated CTI levels are significantly associated with increased incidence and mortality of cardiovascular diseases, indicating that CTI may serve as a strong predictor of cardiovascular outcomes (16).

However, the relationship between CTI and the prognosis of patients with STEMI remains unclear. Therefore, the present study aimed to investigate the association between CTI and the risk of cardiovascular adverse events in patients with STEMI and to evaluate its potential value as a prognostic biomarker for risk stratification in this population.

## 2 Materials and methods

### 2.1 Study population

Patients with ST-segment elevation myocardial infarction (STEMI) admitted to the Department of Cardiology at Jinhua Municipal Central Hospital between January 1, 2020 and May 31, 2023 were retrospectively screened.

The inclusion criteria were: (1) diagnosis of STEMI according to the Fourth Universal Definition of Myocardial Infarction (17); (2) admission within 7 days of symptom onset; and (3) age >18 years.

**Figure 1:**
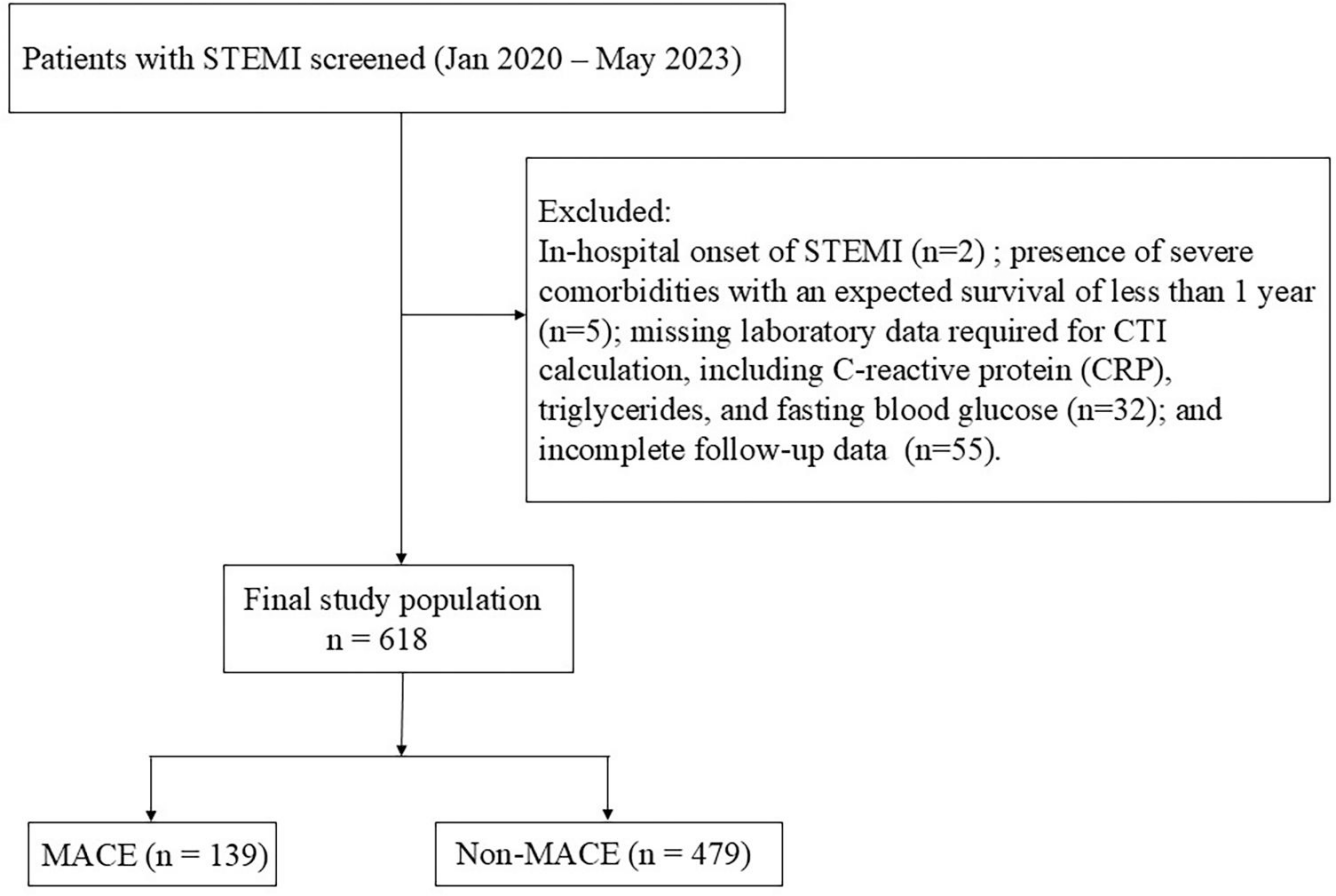
Flowchart of patient selection and study design.

The exclusion criteria were: (1) in-hospital onset of STEMI; (2) presence of severe comorbidities with an expected survival of less than 1 year; (3) missing laboratory data required for CTI calculation, including C-reactive protein (CRP), triglycerides, and fasting blood glucose; and (4) incomplete follow-up data. Finally, a total of 618 patients were included in the analysis (Figure 1).

This study was approved by the Medical Ethics Committee of Jinhua Municipal Central Hospital [Approval No.: (Medical) 2019-Ethics Review-42], and written informed consent was obtained from all participants.

### 2.2 Data collection

Baseline clinical data were collected from the hospital medical record system, including age, sex, smoking status, alcohol consumption, and medical history (hypertension and diabetes).

Laboratory parameters were obtained from the first fasting blood test after admission, including fasting blood glucose, triglycerides, C-reactive protein (CRP), glycated hemoglobin (HbA1c), renal function indicators, lipid profiles, etc. Cardiac biomarkers were measured at admission and repeatedly during hospitalization according to standard clinical practice (typically at 3–6 h intervals), and the peak value was used for analysis.

Echocardiographic parameters, particularly left ventricular ejection fraction (LVEF), were also recorded.

### 2.3 Follow-up and outcomes

Patients were routinely followed up at 1 month, 6 months, and 12 months after discharge through telephone interviews, outpatient visits, and review of rehospitalization records. Additional follow-up information obtained outside these predefined time points was also recorded.

The primary endpoint was the occurrence of major adverse cardiovascular events (MACE) within 365 days after discharge. MACE was defined as a composite of acute heart failure, in-stent thrombosis, stroke, recurrent myocardial infarction, malignant arrhythmia, and cardiac death.

Event time was defined as the interval from PCI to the first occurrence of MACE or the last available follow-up. Patients without MACE during follow-up were treated as censored observations at the time of the last follow-up visit. Patients who did not complete follow-up or were lost to follow-up were excluded according to the predefined exclusion criteria.

### 2.4 Calculation of CTI

CRP and TyG were used to reflect patients' inflammation and IR status, respectively. The CRP-TyG index (CTI) was composed of CRP and TyG (18). The CTI index was calculated according to the established equation:

$$CTI\;=\;0.412\times ln(\mathrm{CRP})+\;ln\left(\frac{\mathrm{TG}\times \;\mathrm{FPG}\;}{2}\right)$$

### 2.5 Missing data handling

Missing data were assessed for all variables prior to analysis. Left ventricular ejection fraction (LVEF) was missing in 44 patients (7.1%). Missingness for all other variables was less than 5%.

Given the clinical importance of LVEF, missing values were handled using multiple imputation. Multiple imputation by chained equations was performed to generate imputed datasets, and results were pooled according to Rubin's rules.

Sensitivity analyses based on complete cases yielded consistent results.

### 2.6 Statistical analysis

All statistical analyses were performed using R software (version 4.5.2). Continuous variables were expressed as mean ± standard deviation or median with interquartile range, as appropriate, and categorical variables were presented as numbers and percentages. Comparisons between patients with and without MACE were performed using Student's *t*-test or the Mann–Whitney U test for continuous variables, and the chi-square test or Fisher's exact test for categorical variables. Missing data were handled using multiple imputation by chained equations.

Feature selection was performed using the Boruta algorithm to identify variables potentially associated with MACE. Univariable Cox proportional hazards regression analysis was first conducted to evaluate the association between baseline variables and 1-year MACE. Variables selected based on univariable analysis, Boruta feature selection, and clinical relevance were subsequently included in multivariable Cox regression models. Event time was defined as the interval from PCI to the first occurrence of MACE or the last available follow-up. Patients without MACE during follow-up were treated as censored observations.

Two Cox models were constructed to evaluate the incremental prognostic value of CTI. Model 1 included age, sex, diastolic blood pressure, heart rate, history of diabetes, Killip class, LDL cholesterol, and LVEF. Model 2 included all variables in Model 1 plus CTI. Hazard ratios (HRs) and 95% confidence intervals (CIs) were reported. The proportional hazards assumption was assessed using Schoenfeld residual–based tests.

The predictive performance of the models was evaluated using 365-day time-dependent receiver operating characteristic (ROC) curve analysis, and differences in area under the curve (AUC) values were compared using the DeLong test. Decision curve analysis (DCA) was performed to evaluate the clinical net benefit of models with and without CTI. MACE components were compared across CTI quartiles using the chi-square test or Fisher's exact test, as appropriate. Subgroup analysis was performed according to diabetes status, and interaction was tested by including a multiplicative interaction term between CTI and diabetes status in the Cox model.

A two-sided *P* value < 0.05 was considered statistically significant.

## 3 Results

### 3.1 Baseline characteristics of the study population

A total of 618 patients were included in this study, among whom 139 (22.5%) experienced major adverse cardiovascular events (MACE) during follow-up (Table 1). Compared with patients without MACE, those who developed MACE were older and had a higher proportion of males (both *P* < 0.05). In addition, patients in the MACE group had higher heart rate, lower body mass index (BMI), and lower diastolic blood pressure, as well as elevated levels of HbA1c, creatinine, and uric acid, whereas low-density lipoprotein cholesterol (LDL-C) and total cholesterol levels were lower (all *P* < 0.05). Regarding cardiac function, patients who developed MACE had lower left ventricular ejection fraction (LVEF) and were more likely to present with higher Killip class (both *P* < 0.001). CTI levels were also significantly higher in the MACE group (*P* < 0.001). Furthermore, patients with MACE had a higher prevalence of diabetes, hypertension, and alcohol consumption history. These patients were also more likely to receive diuretics and to have multivessel coronary artery disease (both *P* < 0.001).

**Table 1:** Baseline characteristics of the study population.

Variable		Total (*n* = 618)	Non-MACE (*n* = 479)	MACE (*n* = 139)	*p*-value
Demographic characteristics					
Age, years		64.61 (13.78)	62.89 (13.02)	70.57 (14.68)	<0.001
Sex, *n* (%)					0.001
	Male	506 (81.88%)	405 (84.55%)	101 (72.66%)	
	Female	112 (18.12%)	74 (15.45%)	38 (27.34%)	
Vital signs					
BMI, kg/m²		23.92 (2.57)	24.08 (2.44)	23.37 (2.90)	0.009
Systolic BP, mmHg		134.06 (25.44)	134.67 (24.67)	131.93 (27.90)	0.296
Diastolic BP, mmHg		83.86 (16.81)	84.70 (16.21)	80.95 (18.52)	0.032
Heart rate, bpm		79.92 (18.89)	77.68 (16.49)	87.64 (24.01)	<0.001
Respiratory rate, breaths/min		19.47 (3.36)	19.43 (3.38)	19.60 (3.28)	0.610
Laboratory parameters					
Creatinine, μmol/L		82.69 (44.92)	80.22 (42.89)	91.21 (50.53)	0.021
HbA1c, %		6.26 (1.36)	6.19 (1.30)	6.52 (1.52)	0.022
Peak troponin I, ng/mL		147.67 (225.65)	153.10 (238.49)	128.95 (173.76)	0.189
Peak CK-MB, U/L		255.00 (385.76)	253.11 (421.79)	261.49 (221.51)	0.756
Total cholesterol, mmol/L		4.46 (1.14)	4.52 (1.14)	4.29 (1.11)	0.035
HDL, mmol/L		1.06 (0.27)	1.05 (0.26)	1.09 (0.31)	0.179
LDL, mmol/L		2.96 (0.89)	3.02 (0.90)	2.76 (0.79)	<0.001
Uric acid, μmol/L		343.59 (106.28)	335.53 (98.91)	371.37 (124.94)	0.002
Cardiac function					
LVEF, %		57.95 (9.58)	59.48 (8.58)	52.65 (10.90)	<0.001
Killip Class, *n* (%)					<0.001
	I	490 (79.29%)	410 (85.59%)	80 (57.55%)	
	II	59 (9.55%)	37 (7.72%)	22 (15.83%)	
	III	15 (2.43%)	6 (1.25%)	9 (6.47%)	
	IV	54 (8.74%)	26 (5.43%)	28 (20.14%)	
CTI					
		9.51 (1.00)	9.38 (0.93)	9.96 (1.08)	<0.001
Medical history					
Hypertension, *n* (%)		332 (53.72%)	245 (51.15%)	87 (62.59%)	0.017
Diabetes, *n* (%)		120 (19.42%)	77 (16.08%)	43 (30.94%)	<0.001
Smoking, *n* (%)		361 (58.41%)	288 (60.13%)	73 (52.52%)	0.109
Alcohol Drinking, *n* (%)		136 (22.01%)	93 (19.42%)	43 (30.94%)	0.004
Medications					
Aspirin, *n* (%)		618.00 (100.00%)	479.00 (100.00%)	139.00 (100.00%)	
Clopidogrel, *n* (%)		397.00 (64.24%)	293.00 (61.17%)	104.00 (74.82%)	0.003
Ticagrelor, *n* (%)		220.00 (35.60%)	185.00 (38.62%)	35.00 (25.18%)	0.004
Beta Blocker, *n* (%)		426 (68.93%)	336 (70.15%)	90 (64.75%)	0.226
ACEI or ARB, *n* (%)		421 (68.12%)	327 (68.27%)	94 (67.63%)	0.886
Statin type, *n* (%)					0.286
	rosuvastatin	234 (37.86%)	176 (36.74%)	58 (41.73%)	
	atorvastatin	384 (62.14%)	303 (63.26%)	81 (58.27%)	
LMWH, *n* (%)		423 (68.45%)	323 (67.43%)	100 (71.94%)	0.314
Diuretics, *n* (%)		201 (32.52%)	122 (25.47%)	79 (56.83%)	<0.001
PCI-related characteristics					
Infarct-related artery, *n* (%)					0.762
	Left anterior descending artery	316 (51.13%)	244 (50.94%)	72 (51.80%)	
	Left circumflex artery	75 (12.14%)	60 (12.53%)	15 (10.79%)	
	Right coronary artery	222 (35.92%)	172 (35.91%)	50 (35.97%)	
	Left main artery	5 (0.81%)	3 (0.63%)	2 (1.44%)	
Number of diseased vessels, *n* (%)					<0.001
	Single-vessel disease	314 (50.81%)	259 (54.07%)	55 (39.57%)	
	Double-vessel disease	156 (25.24%)	125 (26.10%)	31 (22.30%)	
	Triple-vessel disease	148 (23.95%)	95 (19.83%)	53 (38.13%)	
Number of stents, *n* (%)					0.488
	1	494 (79.94%)	384 (80.17%)	110 (79.14%)	
	2	107 (17.31%)	84 (17.54%)	23 (16.55%)	
	3	16 (2.59%)	10 (2.09%)	6 (4.32%)	
	≥4	1 (0.16%)	1 (0.21%)	0 (0.00%)	
Average stent diameter, mm (mean ± SD)		3.07 (0.38)	3.08 (0.39)	3.06 (0.38)	0.612
Total stent length, mm (mean ± SD)		29.34 (12.79)	28.98 (12.60)	30.57 (13.41)	0.214
Post-PCI TIMI flow grade 3, *n* (%)		553.00 (89.48%)	426.00 (88.94%)	127.00 (91.37%)	0.411

MACE, major adverse cardiovascular events; BMI, body mass index; BP, blood pressure; HbA1c, glycated hemoglobin; CK-MB, creatine kinase-MB; HDL, high-density lipoprotein; LDL, low-density lipoprotein; LVEF, left ventricular ejection fraction; CTI, C-reactive protein–triglyceride glucose index; LMWH, low-molecular-weight heparin; ACEI, angiotensin-converting enzyme inhibitor; ARB, angiotensin receptor blocker.

### 3.2 Feature selection using the Boruta algorithm

**Figure 2:**
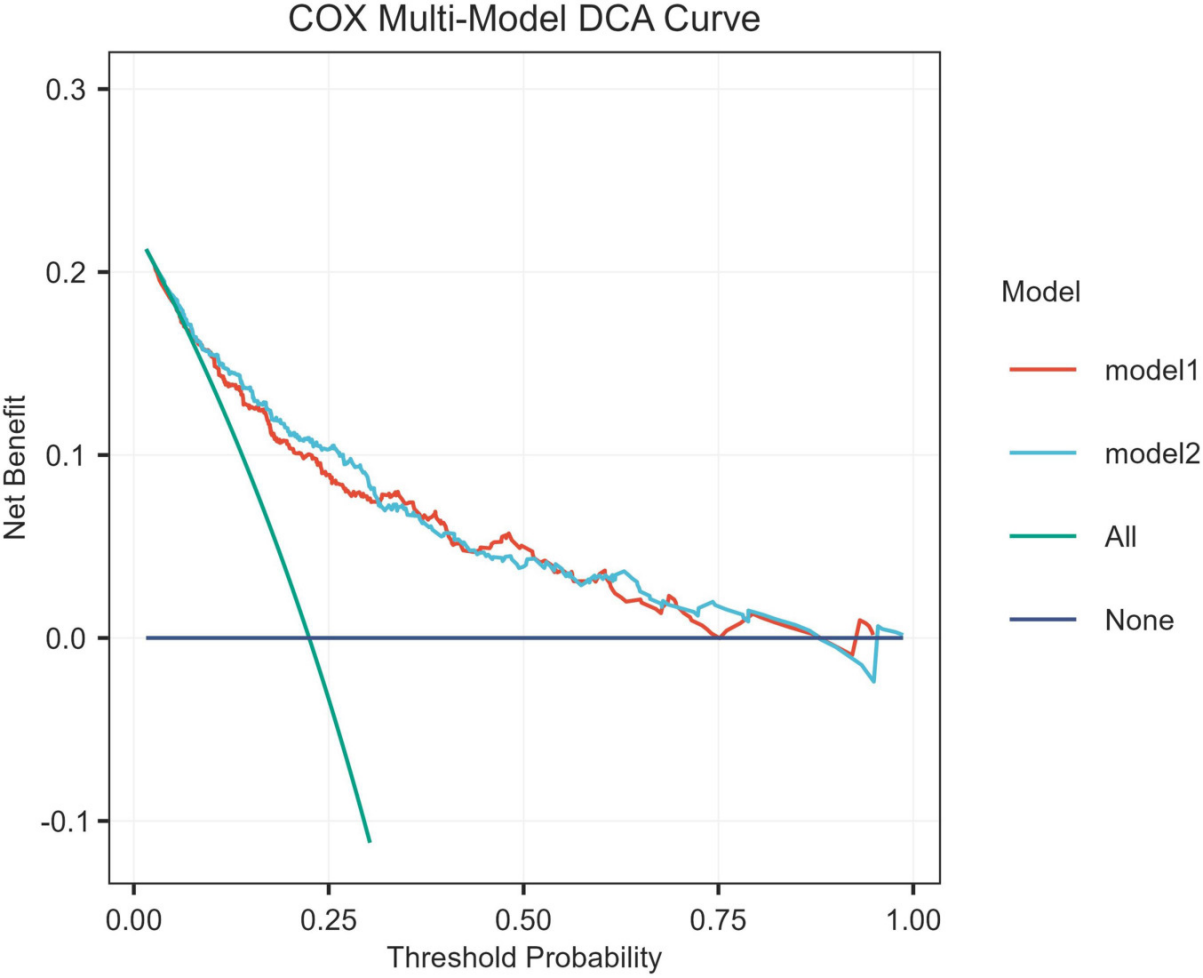
Variable importance plot based on the Boruta algorithm.

Feature selection was performed using the Boruta algorithm, and the results are presented in Figure 2. A total of 16 variables were confirmed as important predictors of MACE, including LVEF, CTI, heart rate, age, Killip class, diuretic use, diastolic blood pressure, BMI, LDL, creatinine, history of diabetes, HbA1c, uric acid, sex, alcohol drinking, sex and total cholesterol.

### 3.3 Multivariable Cox regression and model performance

**Figure 3:**
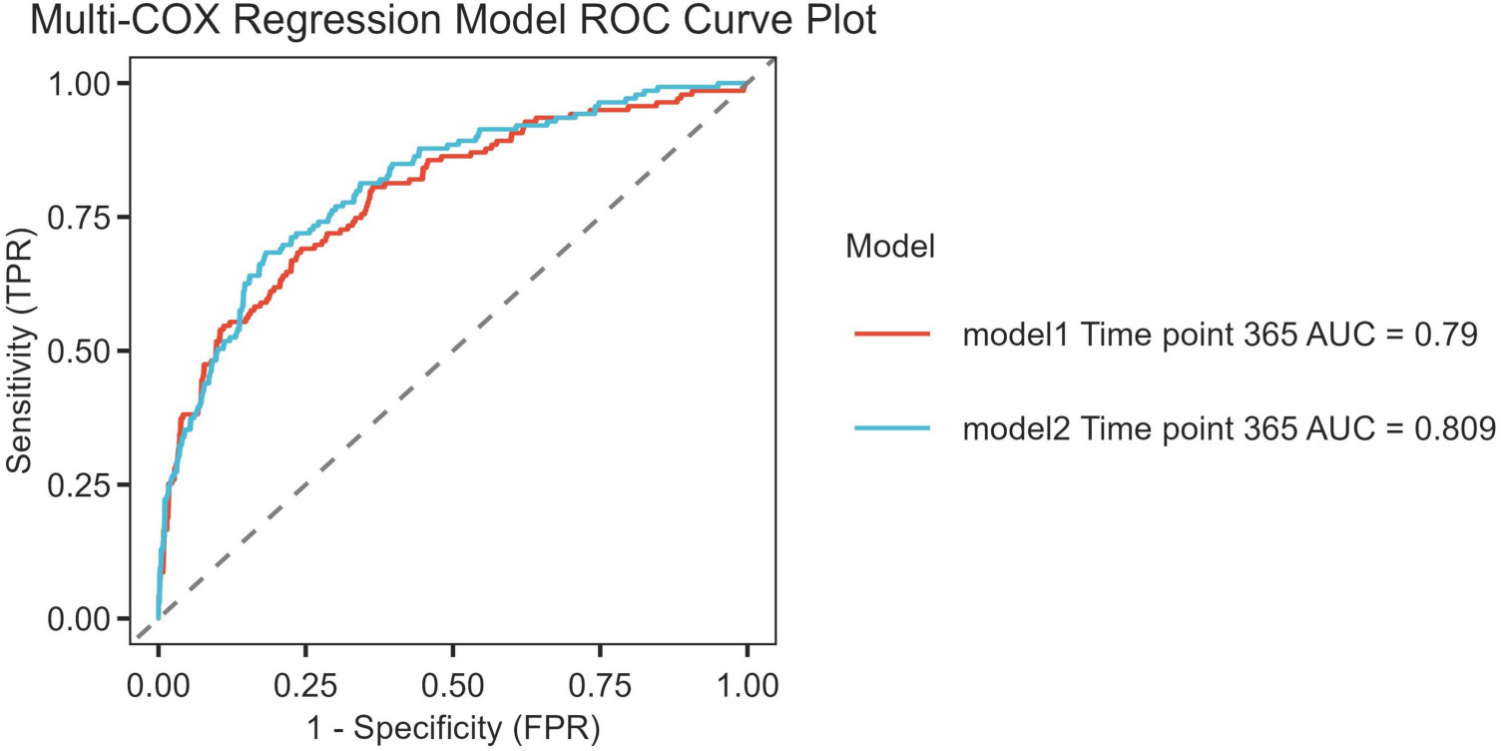
Time-dependent ROC curves of Cox regression models for predicting 1-year MACE.

**Figure 4:**
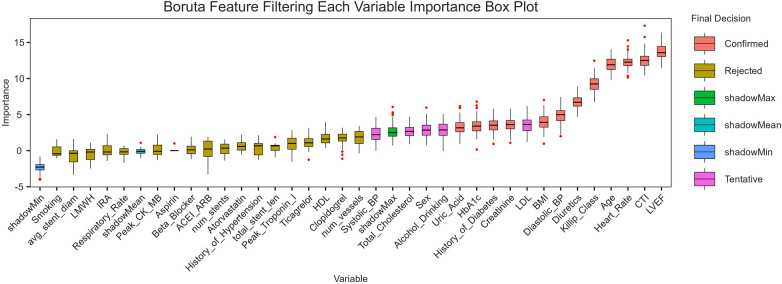
Decision curve analysis of Cox regression models for predicting 1-year MACE.

Based on univariate analysis (Supplementary Table S1), Boruta feature selection, and clinical relevance, variables were selected for multivariable Cox regression. CTI remained independently associated with MACE (HR = 1.443, 95% CI: 1.191–1.748, *P* < 0.001), along with age, heart rate, LVEF, Killip class, and diabetes (Table 2). The addition of CTI to the baseline model modestly but significantly improved discrimination (AUC: 0.809 vs. 0.790; DeLong test *P* = 0.0423) (Figure 3). Decision curve analysis further demonstrated that the model including CTI provided a higher net clinical benefit across a range of threshold probabilities (Figure 4).

**Table 2:** Multivariable Cox regression analysis for MACE.

Variable	Model 1 HR (95% CI)	*P* value	Model 2 HR (95% CI)	*P* value
CTI	—	—	1.443 (1.191–1.748)	<0.001
Age	1.019 (1.006–1.033)	0.004	1.022 (1.009–1.035)	0.001
Diastolic BP	0.990 (0.978–1.001)	0.082	0.988 (0.977–1.000)	0.049
Heart rate	1.010 (1.002–1.018)	0.020	1.009 (1.001–1.017)	0.038
Diabetes	1.719 (1.179–2.506)	0.005	1.418 (0.960–2.095)	0.079
Killip class II	2.326 (1.436–3.767)	0.001	2.380 (1.468–3.858)	<0.001
Killip class III	2.390 (1.134–5.036)	0.022	2.060 (0.964–4.405)	0.062
Killip class IV	2.514 (1.565–4.037)	<0.001	2.327 (1.447–3.744)	<0.001
LDL	0.805 (0.653–0.992)	0.041	0.811 (0.662–0.994)	0.043
LVEF	0.953 (0.936–0.969)	<0.001	0.960 (0.943–0.978)	<0.001
Sex	1.171 (0.783–1.751)	0.442	1.159 (0.778–1.726)	0.468

Model 1 was adjusted for age, sex, diastolic blood pressure, heart rate, history of diabetes, Killip class, low-density lipoprotein (LDL), and left ventricular ejection fraction (LVEF). Model 2 included all variables in Model 1 with the additional adjustment for CTI.

HR, hazard ratio; CI, confidence interval; CTI, C-reactive protein–triglyceride glucose index; LDL, low-density lipoprotein; LVEF, left ventricular ejection fraction; BP, blood pressure; MACE, major adverse cardiovascular events.

### 3.4 Distribution of MACE components across CTI quartiles

The incidence of MACE increased significantly across CTI quartiles, from 13.5% in Q1 to 38.7% in Q4 (*P* < 0.001) (Table 3). Among individual components, acute heart failure showed a significant increasing trend across CTI quartiles (*P* < 0.001), with incidence rising from 7.1% in Q1 to 25.2% in Q4. In contrast, other MACE components did not differ significantly across CTI quartiles (all *P* > 0.05), although numerically higher rates were observed in the highest quartile. These findings suggest that the association between CTI and MACE may be primarily driven by acute heart failure.

**Table 3:** Distribution of MACE and its components across CTI quartiles.

Outcomes	Total *n* (%)	Q1 *n* = 155	Q2 *n* = 154	Q3 *n* = 154	Q4 *n* = 155	*P* value
MACE	139 (22.5)	21 (13.5)	23 (14.9)	35 (22.7)	60 (38.7)	<0.001
Cardiac Death	21 (3.4)	5 (3.2)	4 (2.6)	5 (3.2)	7 (4.5)	0.822
Acute Heart Failure	86 (13.9)	11 (7.1)	12 (7.8)	24 (15.6)	39 (25.2)	<0.001
Recurrent Myocardial Infarction	17 (2.8)	2 (1.3)	3 (1.9)	3 (1.9)	9 (5.8)	0.061
In-stent Thrombosis	2 (0.3)	2 (1.3)	0 (0.0)	0 (0.0)	0 (0.0)	0.112
Malignant Arrhythmia	7 (1.1)	1 (0.6)	3 (1.9)	2 (1.3)	1 (0.6)	0.657
Stroke	6 (1.0)	0 (0.0)	1 (0.6)	1 (0.6)	4 (2.6)	0.110

Data are presented as *n* (%). *P* values were calculated using the chi-square test or Fisher's exact test, as appropriate. Q1–Q4 represent quartiles of the C-reactive protein–triglyceride–glucose index (CTI). MACE included acute heart failure, in-stent thrombosis, stroke, recurrent myocardial infarction, malignant arrhythmia, and cardiac death.

CTI, C-reactive protein–triglyceride glucose index; MACE, major adverse cardiovascular events.

### 3.5 Subgroup analysis

Subgroup analysis demonstrated that the association between CTI and MACE was consistent across diabetes status. CTI remained significantly associated with MACE in both diabetic and non-diabetic patients, with no significant interaction observed (*P* for interaction = 0.504) (Table 4).

**Table 4:** Subgroup analyses and interaction tests for the association between CTI and MACE.

Subgroup	HR (95% CI)	*P* value
Overall		
CTI	1.443 (1.191–1.748)	<0.001
Diabetes		
No	1.324 (1.043–1.692)	0.015
Yes	1.725 (1.212–2.457)	0.003
Interaction (CTI × Diabetes)	—	0.504

Hazard ratios (HRs) and 95% confidence intervals (CIs) were estimated using Cox proportional hazards regression models. The overall estimate was derived from the primary multivariable Cox model. Subgroup-specific estimates were obtained from stratified Cox regression analyses according to diabetes status. *P* for interaction was calculated by including a multiplicative interaction term between CTI and diabetes status in the Cox model.

CTI, C-reactive protein-triglyceride glucose index; HR, hazard ratio; CI, confidence interval.

### 3.6 Sensitivity analyses

To address potential confounding by disease severity and treatment indication, several clinically driven sensitivity analyses were performed (Supplementary Table S2). First, after excluding Killip class from the model, CTI remained independently associated with MACE (HR = 1.472, 95% CI: 1.218–1.779, *P* < 0.001). Second, after additionally adjusting for diuretic use, CTI remained a significant predictor (HR = 1.418, 95% CI: 1.168–1.722, *P* < 0.001). Third, in a parsimonious model including only core clinical variables, CTI was still independently associated with MACE (HR = 1.416, 95% CI: 1.172–1.709, *P* < 0.001). Overall, the association between CTI and MACE remained robust across all sensitivity analyses.

## 4 Discussion

In this study, we investigated the association between CTI and 1-year MACE in STEMI patients undergoing PCI using Cox regression–based survival analysis. CTI was identified as an important variable by Boruta feature selection and remained independently associated with MACE after multivariable adjustment. Although the addition of CTI to the baseline clinical model provided only modest improvement in discrimination and clinical net benefit, further analysis demonstrated that the association between CTI and adverse outcomes was mainly driven by acute heart failure events. In addition, the association between CTI and MACE remained consistent across diabetes subgroups and generally consistent across multiple sensitivity analyses. These findings suggest that CTI may reflect inflammatory–metabolic mechanisms related to post-infarction ventricular dysfunction in STEMI patients after PCI.

The C-reactive protein–triglyceride glucose index (CTI) is a novel composite biomarker integrating systemic inflammation and metabolic dysfunction. By combining C-reactive protein (CRP), triglycerides, and fasting glucose, CTI reflects both inflammatory activation and insulin resistance, two key pathophysiological processes involved in atherosclerosis progression, myocardial injury, and adverse ventricular remodeling after STEMI. These mechanisms may partly explain the observed association between elevated CTI levels and adverse cardiovascular outcomes in patients undergoing PCI. Previous large-scale cohort studies have also demonstrated significant associations between CTI and cardiovascular disease, stroke, and cardiovascular mortality (16, 19, 20). Our findings further extend these observations to STEMI patients after PCI and suggest that CTI may be particularly associated with acute heart failure events.

Several findings in this study deserve further discussion. First, the lower LDL-C levels observed in patients with MACE should be interpreted cautiously. Although elevated LDL-C is a well-established causal risk factor for atherosclerosis, lower LDL-C levels at admission in acute myocardial infarction may reflect the so-called “lipid paradox.” This phenomenon may be related to reverse causality, acute-phase lipid changes, stronger systemic inflammation, poorer nutritional status, or frailty in higher-risk patients rather than a truly protective effect of LDL-C (21, 22). Therefore, the inverse association observed in this study does not contradict the established benefit of lipid-lowering therapy after myocardial infarction.

Second, alcohol consumption showed a positive association with MACE risk. However, this finding should be interpreted cautiously because detailed information regarding alcohol intake patterns and quantity was unavailable in the present study. Previous studies have suggested that excessive alcohol consumption may contribute to adverse cardiovascular outcomes through mechanisms including arrhythmia, hypertension, myocardial injury, and progressive deterioration of cardiac function, particularly progression of heart failure (23, 24).

Third, the observed association between diuretic use and increased MACE risk likely reflects confounding by indication, as diuretics are more frequently prescribed in patients with impaired cardiac function or heart failure. This interpretation is further supported by the component analysis of MACE in the present study, in which the association between CTI and adverse outcomes appeared to be mainly driven by acute heart failure events. Similarly, lower LVEF and higher Killip class were also strongly associated with MACE, suggesting that CTI may be more closely related to ventricular dysfunction and heart failure progression rather than recurrent ischemic events alone.

At the same time, to address potential concerns regarding confounding, particularly from variables reflecting disease severity or treatment indication, multiple sensitivity analyses were conducted. The association between CTI and MACE remained generally consistent after excluding Killip class, additionally adjusting for diuretic use, and using a simplified clinically driven model. These findings support the stability of the observed association between CTI and MACE.

This study has several strengths. We applied a combination of machine learning–based feature selection and clinically guided modeling to improve variable selection and reduce potential overfitting. In addition, subgroup analysis and multiple sensitivity analyses were performed to further evaluate the stability of the association between CTI and MACE.

Several limitations should be acknowledged. First, this was a single-center retrospective study, which may limit the generalizability of the findings and introduce selection bias. Second, although multiple covariates were adjusted, residual confounding cannot be completely excluded, particularly from unmeasured factors such as medication adherence and lifestyle variables. Third, some variables, including alcohol consumption, were not quantified in detail, which may affect the interpretation of their associations. Fourth, missing data, particularly for LVEF, were handled using multiple imputation, which may have introduced additional uncertainty. Fifth, although CTI showed an independent association with MACE, the improvement in discrimination and clinical net benefit after adding CTI to the baseline model was relatively modest. Finally, causal inference cannot be established due to the observational design of the study.

From a clinical perspective, CTI represents a simple and readily available biomarker derived from routinely measured laboratory parameters. Because CTI integrates both inflammatory and metabolic disturbances, it may help identify STEMI patients at increased risk of post-infarction ventricular dysfunction and acute heart failure events after PCI. However, given the modest improvement in predictive performance observed in the present study, CTI should currently be considered a supplementary marker rather than a standalone clinical prediction tool. Further prospective multicenter studies are warranted to validate these findings and clarify the clinical role of CTI in STEMI patients after PCI.

## 5 Conclusion

In conclusion, CTI, a novel marker integrating inflammatory and metabolic pathways, was independently associated with the risk of MACE in patients with STEMI following PCI. The observed association appeared to be mainly driven by acute heart failure events, suggesting a potential link between CTI and post-infarction ventricular dysfunction. Although CTI provided only modest incremental prognostic information beyond conventional clinical factors, its simplicity and accessibility may support its use as a supplementary marker in clinical assessment. Further prospective multicenter studies are warranted to validate these findings and clarify the underlying mechanisms.

## Data Availability

The raw data supporting the conclusions of this article will be made available by the authors, without undue reservation.
