# Supplementary material for: Prognostic value of CTI for major adverse cardiovascular events in patients With ST-elevation myocardial infarction after primary percutaneous coronary intervention
Source: Front Cardiovasc Med. 2026 May 26;13:1832830. doi: 10.3389/fcvm.2026.1832830 (PMC13246453; doi:10.3389/fcvm.2026.1832830)
Supplement: Supplementary file 1 [file Table1.docx]

**Supplementary Table 1. Univariable Cox regression analysis for predictors of MACE**

|  | **HR (95% CI)** | **P value** |
| --- | --- | --- |
| **Continuous variables** |  |  |
| Age (per year) | 1.036 (1.024–1.047) | <0.001 |
| BMI (kg/m²) | 0.908 (0.850–0.970) | 0.004 |
| Diastolic BP (mmHg) | 0.987 (0.977–0.997) | 0.014 |
| Heart rate (bpm) | 1.023 (1.015–1.031) | <0.001 |
| Creatinine (μmol/L) | 1.003 (1.000–1.005) | 0.020 |
| CTI | 1.663 (1.410–1.960) | <0.001 |
| HbA1c (%) | 1.127 (1.023–1.242) | 0.015 |
| LDL (mmol/L) | 0.719 (0.588–0.880) | 0.001 |
| Uric acid (μmol/L) | 1.002 (1.001–1.004) | <0.001 |
| Total cholesterol (mmol/L) | 0.844 (0.725-0.982) | 0.028 |
| LVEF (%) | 0.938 (0.923–0.953) | <0.001 |
|  |  |  |
| **Categorical variables** |  |  |
| Sex (female vs male) | 1.816 (1.250–2.637) | 0.002 |
| Diabetes (yes vs no) | 2.046 (1.428–2.932) | <0.001 |
| Hypertension (yes vs no) | 1.519 (1.077–2.142) | 0.017 |
| Alcohol drinking (yes vs no) | 1.706 (1.191–2.445) | 0.004 |
| Diuretics use (yes vs no) | 3.310 (2.364–4.633) | <0.001 |
|  |  |  |
| **Killip class (reference: class I)** |  |  |
| Class II | 2.614 (1.630–4.191) | <0.001 |
| Class III | 4.931 (2.472–9.839) | <0.001 |
| Class IV | 4.178 (2.714–6.431) | <0.001 |
|  |  |  |

Abbreviations: HR, hazard ratio; CI, confidence interval; BP, blood pressure; LDL, low-density lipoprotein; LVEF, left ventricular ejection fraction; CTI, C-reactive protein–triglyceride–glucose index; MACE, major adverse cardiovascular events.

Continuous variables are presented per unit increase. Categorical variables were analyzed using appropriate reference categories as indicated.

**Supplementary Table 2. Sensitivity analyses of the association between CTI and MACE**

| **Model** | **Adjustment** | **HR (95% CI)** | **P value** |
| --- | --- | --- | --- |
| Main model | Full adjustment | 1.443 (1.191–1.748) | <0.001 |
| Sensitivity 1 | Excluding Killip class | 1.472 (1.218–1.779) | <0.001 |
| Sensitivity 2 | + Diuretics | 1.418 (1.168–1.722) | <0.001 |
| Sensitivity 3 | Clinical core model | 1.416 (1.172–1.709) | <0.001 |

Notes: Hazard ratios (HRs) and 95% confidence intervals (CIs) were estimated using multivariable Cox proportional hazards models.

The main model was constructed based on variables selected from univariate analysis, Boruta feature selection, and clinical relevance.

To address potential confounding by disease severity and treatment indication, three sensitivity analyses were performed:

(1) excluding Killip class, a marker of cardiac dysfunction;

(2) additionally adjusting for diuretic use, which may reflect treatment indication;

(3) using a parsimonious model including core clinically relevant variables (age, sex, LVEF, Killip class, and CTI).

Abbreviations: CTI, C-reactive protein–triglyceride–glucose index; MACE, major adverse cardiovascular events; HR, hazard ratio; CI, confidence interval; LVEF, left ventricular ejection fraction; LDL, low-density lipoprotein cholesterol.
